# A Case of Adult-onset Henoch-Schönlein Purpura Triggered by Fire Ants

**DOI:** 10.7759/cureus.7341

**Published:** 2020-03-20

**Authors:** Samia Mazumder, Mia Ma, Michele Champigny, Adewunmi Adeyemo

**Affiliations:** 1 Dermatology, Wayne State University School of Medicine, Detroit, USA; 2 Surgery, Wayne State University School of Medicine, Detroit, USA; 3 Surgery, Beaumont Hospital, Dearborn, USA; 4 Surgery, Beaumont Hospital, Trenton, USA

**Keywords:** henoch schönlein purpura, iga vasculitis, insect, fire ants

## Abstract

Adult-onset IgA vasculitis, also known as Henoch-Schönlein purpura (HSP), is a rare disease that often presents with a non-blanchable, purpuric rash and can simultaneously affect the gastrointestinal, renal and musculoskeletal systems. The etiology of HSP is unknown. It can be triggered by any entity that creates an immunological insult, including medications, infections and vaccines. We describe a unique case of an adult woman who presented with HSP after experiencing multiple insect bites from fire ants and mosquitos while traveling overseas.

## Introduction

Henoch-Schönlein purpura (HSP) is a non-thrombocytopenic, IgA vasculitis that can affect the integumentary, gastrointestinal, musculoskeletal and renal systems. The most common manifestation of HSP, present in almost all of the patients, is a petechial or purpuric rash [1-3]. The rash presents in the lower limbs and buttocks and can extend upwards to involve the trunk and upper extremities. The purpura are often palpable, erythematous and of varying sizes. Skin biopsy and subsequent histopathological analysis demonstrate areas of leukocytoclastic vasculitis, and immunofluorescence shows IgA deposition in vessel walls [4-6].

Although HSP is the most common childhood vasculitis, it can occur at any age. Adult-onset HSP is rare, with an incidence of four per 100,000 [7]. In addition to a purpuric rash, patients with adult-onset HSP often present with abdominal pain, oligoarticular arthralgia and renal involvement. Renal involvement, such as decreasing glomerular filtration rate and creatinine, is a strong prognostic factor indicating poor disease outcome compared to adult-onset HSP patients presenting with little or no renal involvement [8].

The etiology of adult-onset HSP is variable and not well known. There have been several cases of HSP presenting with antecedent immunological insult, such as a bacterial or viral infection. One study by Albaramki showed that 41.9% of patients with HSP presented with a preceding upper respiratory tract infection [1,4]. Incidents of purpura following infection with streptococcus, Epstein-Barr virus (EBV) and varicella have also been documented. In addition, certain medications and vaccines, such as the measles vaccine, have been seen to trigger vasculitic reactions [8-10]. We present the case of an elderly woman who developed an ascending purpuric rash after experiencing multiple fire ant and insect bites while traveling abroad.

## Case presentation

A 63-year-old Caucasian female presented to the emergency department with an ascending, purpuric rash accompanied by bilateral lower extremity edema. While the patient was visiting Lebanon, she experienced several fire ant bites on a daily basis that resulted in a erythematous and papular urticarial rash on her lower extremities associated with myalgia and fatigue. Two weeks after the inoculation, the patient began experiencing dyspnea, melena and polyarticular arthralgia in addition to the myalgia and fatigue from the insect bites. At this time, the urticarial rash had evolved into a diffuse, purpuric rash on her lower extremities. This was the first episode she had experienced these symptoms.

**Figure 1:**
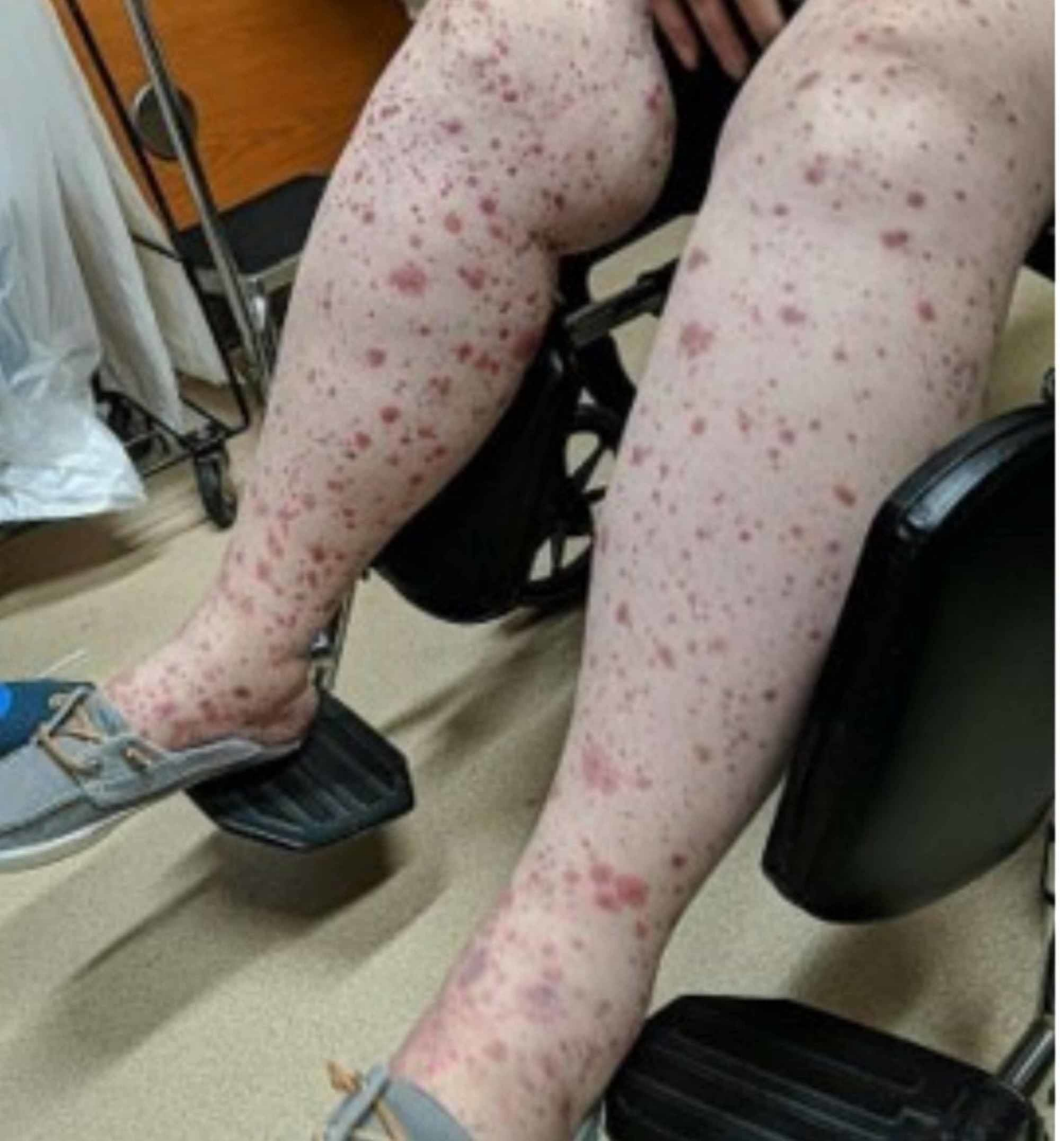
Purpuric rash and edema on bilateral lower extremities

The patient visited a local hospital in Lebanon after the onset of her rash. At the hospital, she was prescribed antibiotics but her symptoms continued to progress. Upon arrival to the United States, she presented to the emergency department with a non-blanchable purpuric, erythematous rash that extended upwards to her lower abdomen (Figure 1). Her lower extremities exhibited bilateral edema and tenderness. She also complained of worsening abdominal pain, fatigue and arthralgia in her ankles, knees and hands.

The patient’s initial laboratory studies demonstrated an elevated white blood cell count (13,000/µL) and absolute neutrophil count (10,600/µL). Her hemoglobin, hematocrit, platelet count, electrolytes, erythrocyte sedimentation rate, kidney function tests and liver function tests were within normal limits. Her antinuclear antibody (ANA), anti-double-stranded DNA (anti-dsDNA), rheumatoid factor and cytoplasmic antineutrophil cytoplasmic antibody (C-ANCA) were negative. Urinalysis was positive for hemoglobinuria with no proteinuria. Imaging studies, including chest x-ray, CT abdomen and pelvis with IV contrast and MRI, were unremarkable.

**Figure 2:**
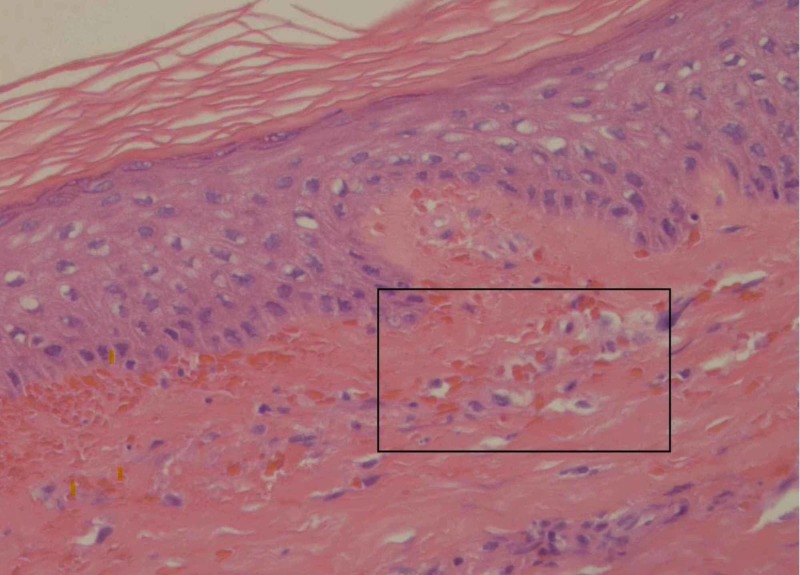
Hematoxylin and eosin stain of punch biopsy The outlined area demonstrates subepidermal, extravasated red blood cells and mild perivascular inflammation with lymphocytes, consistent with purpuric dermatitis.

At the time of her presentation, an infectious etiology was suspected. The patient was started on IV methylprednisolone, IV ceftriaxone and oral metronidazole after admission. Dermatology and general surgery were consulted for evaluation and biopsy of her skin rash. A bedside punch biopsy of two distinct purpuric lesions was performed. Histopathological analysis showed subepidermal proliferation of blood vessels with extravasated red blood cells and mild perivascular inflammation with lymphocytes, neutrophils and eosinophils, consistent with purpuric dermatitis and HSP (Figure 2).

Five days after admission, the patient was discharged with a reducing taper of methylprednisolone and oral metronidazole. At the time of discharge, her purpuric skin rash was visibly reduced. The patient also reported significant improvement of her abdominal pain, arthralgia and fatigue. One month after her initial presentation, the patient was seen in clinic. During the follow-up visit, her symptoms indicating HSP had resolved.

## Discussion

Adult-onset HSP is an uncommon disease with a prevalence of four per 100,000 worldwide [5]. The symptoms of HSP are well described and can include cutaneous purpura, polyarticular arthralgia, hematochezia, abdominal pain and nephritis. However, the etiology of HSP is unknown.

Although the etiology is unknown, previous studies and case reports have associated HSP with infectious agents, such as viruses and bacteria, as well as insect bites. EBV, varicella virus and parvovirus B19 have been implicated in producing leukocytoclastic vasculitis reactions indicating HSP [6,7]. Additionally, bacterial infections such as *Mycoplasma *and *Campylobacter jejuni* have also been directly associated with HSP. These infectious agents are thought to trigger an immunological reaction which results in increased immunocomplex deposition in vessels [8]

HSP has also been associated in patients who suffered a recent insect bite. A case report by Burke and Jellinek describes one of the first and most severe presentations of insect-induced HSP in a child, which necessitated the use of blood transfusions and corticosteroids over the course of 45 days of hospitalization [9]. Additionally, other cases outline a similar association between an insect bite and subsequent HSP. In these cases, the insect bite is usually 5-10 days prior to the development of HSP symptoms [10]. It is hypothesized that an antibody response to the insect antigens and subsequent formation of immune complexes occur during this time gap between inoculation and symptom presentation [10,11].

There have been several studies that support the role of a genetic component on the presentation and severity of HSP in certain population groups. A large case series with Caucasian patients conducted by López-Mejías et al. found that HLA-DRB1*01 was significantly increased in patients with HSP (43%) compared to controls (27%, p<0.001). In addition, the presence of HLA-DRB1*03 is significantly decreased (5.6%) in HSP patients compared to controls (18.2%, p<0.001). These HLA types are believed to influence the immunomodulatory functions of IgA and interleukin 1β. Therefore, HLA molecules can increase one’s susceptibility to developing HSP or play a protective role and decrease the likelihood of disease onset [12].

The clinical category for diagnosis is based on studies conducted by European League Against Rheumatism (EULAR), Pediatric Rheumatology European Society (PRES) and Pediatric Rheumatology International Trials Organization (PRINTO). The category mandates the presence of purpura or petechiae, and the patient must have at least one out of the four other criterion, which includes abdominal pain, histopathological appearance, arthralgia and renal involvement. This criterion boasts a sensitivity of 100% and a specificity of 87% when clinically differentiating HSP from other vasculitis that may present similarly. In addition to the EULAR/PRES/PRINTO clinical criterion for HSP, definitive diagnosis can also be made with histopathological analysis demonstrating leukocytoclastic vasculitis and IgA deposition in blood vessel walls [13].

Since our patient presented to the emergency department two weeks after the onset of her symptoms, it is possible that the histopathological changes described upon biopsy may have been different at the beginning of her disease course. We did not perform a renal biopsy and immunofluorescent studies on her specimens as her condition was already improving after treatment with methylprednisolone. Instead, our patient’s HSP was diagnosed clinically. She demonstrated bilateral purpuric lesions on her extremities and lower abdomen, arthralgia, abdominal pain and hematuria, which is consistent with the aforementioned EULAR/PRES/PRINTO criterion for diagnosing HSP.

HSP treatment is largely centered around glucocorticoids, immunosuppressive agents and angiotensin receptor blocker/angiotensin-converting enzyme inhibitors. It is unclear whether one treatment option offers improved outcomes over others. Corticosteroids have been indicated in the treatment of insect-induced HSP. Corticosteroids treatment in insect-induced HSP can target the hypersensitivity reactions from the insect venom as well as the leukocytoclastic vasculitis from immune complex deposition found in HSP [14,15]. A longitudinal study by Koskela et al. found that both methylprednisolone and cyclosporine A can be used to treat HSP-associated nephritis, but the efficacy of treatment depended on the time from disease onset to treatment, the use of overlapping treatment modalities and the presence of preexisting, comorbid conditions [14,16]. The patient in the clinical vignette was treated with glucocorticoids which significantly improved her symptoms over the course of her hospital stay without any evidence of complications. Since she did not report any underlying renal disease, treatment with cyclosporine A was not considered. 

## Conclusions

This report presents a unique case of adult-onset HSP with multisystem involvement after being exposed to fire ant bites while overseas. Previous studies have suggested a connection between an initial hypersensitivity response to the antigens introduced in an insect bite and the development of HSP. This case stresses the importance of a detailed history and physical exam at the initial presentation as it can improve treatment times and avoid complications of delayed treatment. It is important for clinicians to be aware of the vast variety of triggers that can lead to HSP in order to identify the disease and initiate treatment in a timely manner.

## References

[1] Albaramki J (2016). Henoch-Schonlein purpura in childhood a fifteen-year experience at a tertiary hospital. J Med Liban.

[2] Cui J, Huang LY, Guo J, Wu CR, Zhang B (2019). Diagnosis and treatment of adult mixed-type Henoch-Schonlein purpura. Cent Eur J Immunol.

[3] Jelusic M, Sestan M, Cimaz R, Ozen S (2019). Different histological classifications for Henoch-Schonlein purpura nephritis: which one should be used?. Pediatr Rheumatol Online J.

[4] Jing J, WangY WangY, Xu F (2020). Association of the infectious triggers with childhood Henoch-Schonlein purpura in Anhui province, China. J Infect Public Health.

[5] Jithpratuck W, Elshenawy Y, Saleh H, Youngberg G, Chi DS, Krishnaswamy G (2011). The clinical implications of adult-onset Henoch-Schonelin purpura. Clin Mol Allergy.

[6] Finkel TH, Torok TJ, Ferguson PJ (1994). Chronic parvovirus B19 infetion and systemic necrotising vasculitis: opportunistic infection or aetiological agent?. Lancet.

[7] Lind KM, Gaud J, Pedersen RS (1994). Henoch-Schonlein purpura associated with Campylobacter jejuni enteritis. Scand J Urol Nephrol.

[8] Kraft DM, Mckee D, Scott C (1998). Henoch-Schonlein purpura: a review. Am Fam Physician.

[9] Burke DM, Jellinek HL (1954). Nearly fatal case of Schoenlein-Henoch syndrome following insect bite. AMA Am J Dis Child.

[10] Galvez-Olortegui J, Alvarez-Vargas M, Durand-Vergara J (2015). Henoch Schonlein purpura associated with bee sting: case report. Medwave.

[11] Sharan G, Anand RK, Sinha KP (1966). Schonlein-Henoch syndrome after insect bite. Br Med J.

[12] Lopez-Mejias R, Genre F, Perez BS (2015). Association of HLA-B* 41-02 with Henoch Schonlein purpura (IgA vasculitis) in Spanish individuals irrespective of the HLA-DRB1 statis. Arthritis Res Ther.

[13] Ozen S, Pistorio A, Lusan SM (2010). EULAR/PRINTO/PRES criteria for Henoch-Schönlein purpura, childhood polyarteritis nodosa, childhood Wegener granulomatosis and childhood Takayasu arteritis: Ankara 2008. Part II: final classification criteria. Ann Rheum Dis.

[14] Koskela M, Jahnukainen T, Enden K (2019). Methylprednisolone or cyclosporine in the treatment of Henoch-Schonlein nephritis: a nationwide study. Pediatr Nephrol.

[15] Seigel JM, Brown HE, DiLeo LW (1954). Purpura as a result of insect allergy: treatment with cortisone. Postgrad Med.

[16] Zhong ZX, Tan JX, Tang Y, Tan L, Pei GQ, Qin W (2019). Crescent lesions are not a predictive factor in adult-onset Henoch-Schonlein purpura nephritis. Clin Exp Med.

